# Establishment of pregnant-specific intervals for hemoglobin (Hb) A2, HbF and cut-off points for HbA2 for thalassemia in Chongqing, China

**DOI:** 10.15537/smj.2022.43.4.20210729

**Published:** 2022-04

**Authors:** Lihua Kang, Siwei Yi, Si Tan, Qiuhong Li, Chunli Li

**Affiliations:** *From the Department of Laboratory (Kang, Yi, Tan, Q. Li, C. Li), Chongqing Health Center for Women and Children, Chongqing, China.*

**Keywords:** cut-off points, HbA2, HbF, thalassemia, pregnant females

## Abstract

**Objectives:**

To analyze pregnant-specific intervals for hemoglobin A2 (HbA2), hemoglobin fetal (HbF), and cut-off points of HbA2 for thalassemia in Chongqing, China.

**Methods:**

Between September 2015 and April 2019, the study recruited 10039 individuals of reproductive age. Of which, 4399 healthy normal individuals were selected to determine reference values for HbA2 and HbF. The remaining 5640 individuals suspected of thalassemia were included to explore the cut-off points of HbA2 for thalassemia.

**Results:**

The reference values of HbA2 in males was 2.3-3.2%, in females was 2.1-3.1%, and in pregnant women was 1.9-3.1%. While the reference values of HbF in males was 0.0-0.0%, in females was 0.0-0.9%, and in pregnant women was 0.0-4.3%. Approximately 2.3% cut-off points for pregnant women was determined to be optimal for α-thalassemia screening. In the entire group, 2.5% was best for all α-thalassemia screenings. The cut-off for β-thalassemia screening using HbA2 was 3.2% for the entire group.

**Conclusion:**

The reference interval of HbA2 for pregnant females group was significantly lower than other groups. Therefore, we recommend cut-off points of HbA2 for α-thalassemia at 2.3% for pregnant women. While partitioning was not needed due to gender. Gender and pregnancy had little effect on the cut-off points of HbA2 for β-thalassemia carrier.

**T**halassemia is the most common monogenic inherited disease due to the defective synthesis of hemoglobin (Hb) α and β chains.^1,2^ The prevalence of thalassemia in China is high in Guangdong, Guangxi, Yunnan, Guizhou, Sichuan, and Chongqing.^3-5^ Severe thalassemia can be disabling and even fatal. There are no effective treatments although long-term blood transfusions and chelation therapy are the primary methods used to maintain life.^6,7^ Therefore, determining the genetic risk for the disorder and avoiding pregnancy in cases of high risk is the key way for prevention. This involves the screening of childbearing age couples in progestation and pregnancy.

The primary screening of thalassemia is through blood routine results and capillary electrophoresis that is an important method of detecting Hb variation. Capillary electrophoresis is the current recommended method to screen for thalassemia but is considered only a rescreening method.^8,9^ Genetic screening is used as the definitive diagnostic method. Hemoglobin electrophoresis rescreening is necessary when blood routine analysis results show the mean corpuscular volume (MCV) is <82 fL or the mean corpuscular Hb (MCH) is <28 pg. Hemoglobin A2 (HbA2) levels >3.5% are indicators of β-thalassemia and <2.5% of α-thalassemia.^10,11^ Interestingly, recent studies have reported that HbA2 levels were also linked to pregnancy, geographic region, race, as well as detection method.^12-14^ However, the levels of HbA2 and fetal hemoglobin (HbF) in the population of child-bearing age have not been determined for Chongqing, China. In the current study, we re-assessed the reference values of HbA2 and HbF in the child-bearing age population in Chongqing, China, and evaluated the levels of HbA2 in thalassemia screening.

## Methods

**Figure 1:**
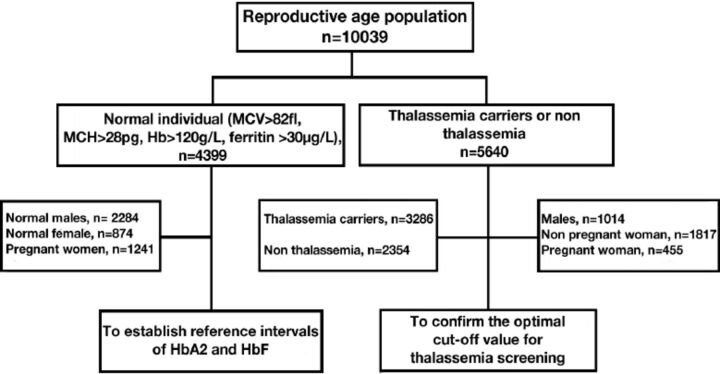
- Schematic diagram of the participating individuals selection process. MCV: mean corpuscular volume, MCH: mean corpuscular hemoglobin, Hb: hemoglobin, HbA2: hemoglobin A2, HbF: fetal hemoglobin

The methodology used in the study has been previously reported, that applied to recruiting participants, sample collection and handling, as well as data analysis.^14,15^ For the selection of reference range, the precautions for grouping, the remove of outliers using Dixon’s rule, and the non-parametric method for statistics calculation were according to Clinical and Laboratory Standards Institute (EP28-A3 criteria 15; Figure 1). Between September 2015 and April 2019, 10039 individuals of reproductive age (targeting those between 20-45 years old) were enrolled in the study. The participants were from Chongqing Province, China, and had been referred to the Chongqing Health Center for Women and Children, Chongqing, China, for variety of reasons, including healthy examination, electrophoresis screening of thalassemia, or genetic testing. The following were exclude: body mass index of >35 kg/m^2^, regular medication for chronic diseases (diabetes, hypertension, hyperlipidemia, allergic diseases, and depression), recently (<15 days) recovering from acute illness, injury, or surgery requiring hospitalization, hepatitis B, hepatitis C, or human immunodeficiency virus carriers. The above results were judged by different senior obstetricians. The included subjects were first screened by blood cell analysis, and the positive ones (MCV: <82 fL, MCH: <28 pg) were re-screened by Hb electrophoresis analysis. Among the indicators detected by electrophoresis analysis for thalassemia (HbA2: >3.5% or <2.5%, and HbF) increased, or abnormal hemoglobin bands appeared. For those with abnormal screening, the peripheral venous blood of these patients was further collected for thalassemia gene detection. Of the 10039 individuals, 4399 were determined to be healthy normal individuals that included 2284 men and 2115 women (1241 non-pregnant and 874 pregnant). These patients were not affected by hypochromic microcytic anemia or other disorders. The remaining 5640 individuals possessed HbA2 levels at <2.5% or >3.5%, and 3286 of them were known and verified thalassemia carriers. This study was approved by the Institutional Ethics Committee of the Chongqing Health Center for Women and Children Hospital, Chongqing, China, and followed the Declaration of Helsinki. All patients were provided with written informed consent prior to their participation in the study.

Venous blood (2 mL) was collected from each subject in K2-dipotassium ethylenediaminetetraacetic acid (EDTA) anticoagulant tubes and hematological analyses were carried out using a Sysmex automated hematological analyzer (Kobe, Japan) within 4 hours after collection. Hemoglobin electrophoresis was carried out using a Sebia capillary electrophoresis instrument (Lisses, France). Total DNA was isolated from blood specimen with DNA purification kit (Promega, Madison, WI, USA) and DNA concentrations were determined by ultraviolet spectroscopy with a NanoDrop ND-1000 instrument (Thermo Fisher, Pittsburg, PA, USA). A commercial real-time polymerase chain reaction kit (Daan, Guangzhou, China) was utilized for screening the presence of genotype 3 α-globin gene deletions (--SEA, -α4.2, -α3.7), 3 α-globin gene mutations (HbCS, HbWS, HbQS) as well as 17 common β-globin gene mutations in -28 (*HBB: c. -78A>G*), -29 (*HBB: c. -79A*>*G*), *CD17* (*HBB: c. 52A>T*), *CD41-42* (*HBB: c. 126-129 delTTCT*), *CD43* (*HBB: c. 130G>T*), β*E* (*HBB: c. 79G>A*), *CD71-72* (*HBB: c. 216_217 insA*), *IVS-II-654* (*HBB: c. 316-197C>T*), *-32* (*HBB: c. -82C>A*), *-30* (*HBB: c. -80T>C*), *CAP* (*HBB: c. -11_8 delAAAC*), *Initiation condon* (*HBB: c. 2T>G*), *CD14-15* (*HBB: c. 45_46 insG*), *CD27-28* (*HBB: c. 84_85 insC*), *IVS-I-1* (*HBB: c. 92+1G>T*), *IVS-I-5* (*HBB: c. 92+5G>C*), and *CD31* (*HBB: c. 94 del C*).

### Statistical analysis

Mean ± standard deviation (SD) were used to present clinical characteristics of the patients. Two tailed t-tests were carried out for comparison between groups with a *p*-value of <0.05 wass considered statistically significant. The Harris and Boyd method was used to evaluate the partition of reference values as previously described.^13,14^ Optimal cut-off points were determined using receiver operating characteristic (ROC) curves that were plotted for males, pregnant women, non-pregnant women, and all groups. The sensitivity, specificity, positive predictive value (PPV) and negative predictive value (NPV), as well as the Youden index were calculated as previously described.^14^ Statistical analysis was carried out using Statistical Package for the Social Sciences, version 25.0 (IBM Corp., Armonk, NY, USA).

## Results

Of 10039 patients selected for the study, 4399 were used for setting control reference values and 5640 for determining cut-off points for thalassemia screening. The reference values for MCV and MCH found in the control group were independents of gender and pregnancy status women. The levels of Hb was >120 µg/L and ferritin was >30 µg/L. In contrast, MCV and MCH levels for α- and β-thalassemia carriers were significantly less than control levels (Table 1).

**Table 1:** - Clinical characteristic of subjects.

Groups	Number of cases	Age (years)	MCV (fl)	MCH (pg)	Hb (g/L)	Ferritin (μg/L)
	Mean±SD					
***Normal subjects for reference intervals***						
Males	2284	32.1±4.2	90.4±3.4	30.6±1.2	158.2±11.3	84.8±16.7
Females	874	30.5±2.9	91.6±3.7	30.4±1.2	136.2±8.7	66.3±25.4
Pregnant women	1241	29.7±4.6	90.2±3.9	30.9±1.4	131.3±8.0	44.5±13.2
***Subjects for cut-off value of HbA2***						
α-thalassemia carriers	1970	31.2±2.3	79.0±8.9	25.4±3.5	130.9±16.5	101.5±20.3
β-thalassemia carriers	1316	28.9±4.7	74.1±11.0	24.4±4.2	125.0±16.6	98.4±24.1
Non thalassemia	2354	29.6±3.9	90.9±4.1	31.3±2.5	147.5±9.8	76.9±18.6

A *p*-value of <0.05 for α-thalassemia and β-thalassemia carriers versus normal group. MCV: mean corpuscular volume, MCH: mean corpuscular hemoglobin, Hb: hemoglobin, HbA2: hemoglobin A2, SD: standard deviation

The Z values determined for HbA2 levels were much lower in normal pregnant women than in males and non-pregnant females, so it was necessary to partition the results into these 2 groups. In addition, HbF levels were not normally distributed using these groupings, so the median values were ranked according to their percentiles as compared with the reference values for HbA2 and HbF. The HbA2 interval for males was 2.3-3.2%, HbA2 interval for females was 2.1-3.1%, and for normal pregnant women was 1.9-3.1%. While these ranges for HbF were 0-0%, 0-0.9%, and 0-4.3% (Table 2).

**Table 2:** - The reference values of normal subjects.

Groups	Percentiles								
	2.5^th^	5^th^	10^th^	25^th^	50^th^	75^th^	90^th^	95^th^	97.5^th^
***HbA2***									
Normal males	2.3	2.4	2.5	2.6	2.8	2.9	3.0	3.1	3.2
Normal females	2.1	2.2	2.3	2.5	2.7	2.8	3.0	3.0	3.1
Normal pregnant women	1.9	2.2	2.3	2.4	2.4	2.7	2.9	3.0	3.1
***HbF***									
Normal males	0.0	0.0	0.0	0.0	0.0	0.0	0.0	0.0	0.0
Normal females	0.0	0.0	0.0	0.0	0.0	0.0	0.5	0.7	0.9
Normal pregnant women	0.0	0.0	0.0	0.0	0.0	1.4	2.8	3.6	4.3

HbA2: hemoglobin A2, HbF: hemoglobin (fetal)

Receiver operating characteristic analyses were carried out using HbA2 levels to determine the optimal cut-off points to generate the best clinical screening efficiency for males, females, and pregnant women in the group that were either known or suspected thalassemia carriers. We found that 2.35% was the optimal cut-off for pregnant women for α-thalassemia screening and this group possessed an area under the ROC curve of 0.63. The total non-control group generated a 2.75% optimal cut-off points for “silent”α-thalassemia, but 2.55% was best when applied to α-thalassemia screening and resulted in an area under the ROC curve of 0.76 (Table 3). The sensitivity for using the HbA2 cut-off points as a screening marker for α-thalassemia was 55.1% in males, 67.9% in non-pregnant women, 71.3% in pregnant women, and 67.9% in all groups. The specificity was 83% in males, 68% in non-pregnant women, 44.9% in pregnant women, and 71.6% in all groups. The PPV for HbA2 at 2.55% was 37.7% in males, 81.8% in non-pregnant women, 74% in pregnant women, and 50.5% in all groups. While NPV value was 40.7% in males, 49.9% in non-pregnant women, 55.7% in pregnant women, and 83.9% in all groups. The Youden indice was 0.381 in males, 0.59 in non-pregnant women, 0.162 in pregnant women, and 0.395 in all groups for the cut off values (Table 4).

**Table 3:** - Optimal cut-off points of hemoglobin A2 used for thalassemia.

Cut-off points	Males	Non pregnant women	Pregnant women	Overall
	Percentages			
***“Silent”*** **α** ***-thalassemia***	2.7	2.7	2.4	2.8
AUC	0.8	0.6	0.5	0.9
**α** ***-thalassemia trait***	2.6	2.6	2.4	2.6
AUC	0.9	0.8	0.8	0.7
**α** ***-thalassemia***	2.5	2.6	2.4	2.6
AUC	0.7	0.7	0.6	0.8
**β** ***-thalassemia***	3.4	3.3	3.3	3.3
AUC	1.0	1.0	1.0	1.0

AUC: area under the curve

**Table 4:** - Screening efficiency of hemoglobin A2 for thalassemia.

Groups	Sensitivity	Specificity	PPV	NPV	Youden index
	Precentage				
***Screening efficiency of HbA2=2.5% for*** **α** ***-thalassemia***					
Males	55.1	83.0	37.7	40.7	0.381
Non pregnant women	67.9	68.0	81.8	49.9	0.359
Pregnant women	71.3	44.9	74.0	55.7	0.162
Overall	67.9	71.6	50.5	83.9	0.395
***Screening efficiency of HbA2=3.2% for*** **β** ***-thalassemia***					
Males	95.2	98.3	96.4	97.5	0.935
Non pregnant women	94.9	98.6	99.0	92.1	0.935
Pregnant women	94.8	99.1	99.0	94.9	0.939
Total	94.8	98.6	98.2	95.9	0.934

PPV: positive predictive value, NPV: negative predictive value, HbA_2_: hemoglobin A2

We also generated ROC curves and cut-off points for screening of β-thalassemia. The HbA2 cut-off points for β-thalassemia was 3.4% in males, 3.3% in non-pregnant women, 3.3% in pregnant women, and 3.3% in the entire group. The area under the ROC curves was 0.98 in males, 0.98 in non-pregnant women, 0.99 in pregnant women, and 0.98 in the entire group (Table 3). Additionally, HbA2 values at 3.3% produced sensitivity and specificity of 94.8% and 98.6% for entire group. The PPVs and NPVs for the cut off values in the latter were 98.2% and 95.9% with a Youden index of 0.934 (Table 4).

Of the 5640 cases of reproductive age population who carried out the genetic testing, 3286 cases were diagnosed with thalassemia, including 937 (28.3%) cases of static α-thalassemia, 909 (28.3%) cases of standard α-thalassemia, 124 (3.7%) cases of non-deletion α-thalassemia, and 1334 (40.4%) cases of β-thalassemia (Table 5).

**Table 5:** - Distribution of genotypes.

Genotype	n (%)
**α** ***-thalassemia***	
-α3.7/αα	767 (23.3)
-α4.2/αα	158 (4.8)
ααCS/αα	63 (1.9)
ααQS/αα	35 (1.1)
ααWS/αα	22 (0.7)
--SEA/αα	889 (27.1)
-α3.7/-α3.7	9 (0.3)
-α3.7/-α4.2	3 (0.1)
--SEA/-α3.7	9 (0.3)
--SEA/-α4.2	2 (0.1)
**β** ***-thalassemia***	
*CD17 (HBB: c.52A>T)*	404 (12.3)
*CD41-42 (HBB: c.126-129 delTTCT)*	348 (10.6)
*IVS-654 (HBB: c.316-197 C>T)*	312 (9.5)
*-28 (HBB: c. -78 A>G)*	79 (2.4)
β *E (HBB: c.79G>A)*	62 (1.9)
*CD71-72 (HBB: c.216_217 insA)*	28 (0.8)
*-29 (HBB: c.-79A>G)*	22 (0.7)
*CD27-28 (HBB: c.84_85 insC)*	43 (1.3)
*CD43(HBB: c.130G>T)*	22 (0.7)
*CD14-15 (HBB: c.45_46 insG)*	4 (0.1)
*CAP (HBB: c. -11_8delAAAC)*	4 (0.1)
α-complex β-thalassemia	3 (0.1)
Total	3286 (-)

## Discussion

Worldwide, Thalassemia is a common genetic disease caused by the deletion or point mutation of the globin gene resulting in insufficient synthesis or deletion of the globin α-chain or β-chain.^16^ A previous child-based study demonstrated that the total frequency of childhood carriers of the disorder was 7.8% in the Chongqing province, China.^4^ Thalassemia has a mutation in the globin gene that results in changes in the composition of hemoglobin, leading to changes at HbF and HbA2 levels.^17^ Therefore, HbF and HbA2 are always considered 2 important detection indicators used in the screening of thalassemia carriers.^18^ However, there were no uniformly recognized reference values for these in Chongqing, China. Gender differences, pregnancy or not, and geographic distribution have all been reported to affect the levels of these 2 indicators. More accurate thalassemia screening values for HbA2 and HbF are needed. Thus, this study was carried out to analysis those levels from individuals of reproductive age in a defined population in Chongqing. It was found from the study that pregnancy had the greatest impact on their levels. Therefore, specific reference ranges and diagnostic values should be established for the pregnancy group.

A total of 10039 patients were enrolled to identify whether gender, age, and pregnancy status were risk factors for thalassemia. We found that the reference values of HbA2 ranged from 2.3-3.2% for males and 2.1-3.1% for non-pregnant females. Gender had little effect on HbA2. Therefore, it is not recommended to divide the reference range according to gender. Our data were similar to those determined for Guizhou and Nanning in China as well as for Malaysia population.^14,19^ Overall, these values were slightly lower than those established by developed countries.^20^ The reference values of HbF ranged from 0-0% in males, 0-0.9% in non- pregnant females, and 0-4.3% in pregnant females, which are consistent with those reported by other large reference laboratories such as Mayo Medical Laboratories.^21^ Furthermore, our data showed that there were significant differences in HbA2 and HbF levels among pregnant group and non-pregnant group. Our study suggests that specific reference ranges for HbA2 and HbF should be set for pregnant women because pregnant group showed significantly lower HbA2 but significantly higher HbF. However, there was no difference in HbA2 and HbF between the pregnant and non-pregnant groups in the Guizhou study.^14^ The reason for the inconsistency with the Guizhou study was mainly due to population differences and regional difference. In contrast with Guizhou, the predominant race in Chongqing is the Han that mirrors that of the general Chinese population. Guizhou area has many ethnic minorities. In addition to this, there are many differences in the sample size of pregnant women.

We also found prominent differences for the reference control intervals of HbF between pregnant and non-pregnant women. A large sample size indicated that level of HbA2 and HbF were different in pregnant goup and non-prgnant group. This difference was most likely the result of HbF release into maternal circulation while differences in HbA2 levels are not fully clear and need further investigation. Given this differences due to pregnancy, a fixed universal reference interval would result in excessive false positive results leading to economic and psychological pressures to be unnecessarily placed on pregnant women.

Hemoglobin A2 is a commonly known index for screening of thalassemia carrier. However, its cut-off points established varied from previous studies.^22^ In addition, there were few extensive studies using HbA2 for screening of thalassemia carrier in the Chinese population. A total of 5640 child-bearing age in Chongqing, China, were enrolled in this study to get HbA2 cut-off points for thalassemia prediction. It was found that pregnancy or not had an obvious influence on the level of HbA2 for α-thalassemia and 2.35% was considered to be an optimal cut off value for pregnant women in Chongqing, China.

We found no significant differences of HbA2 between males and females. Therefore, partitioning was not needed and the same cut-off points for HbA2 can be used because it is gender-independent. We recommend a 2.55% cut-off points for HbA2 for α-thalassemia prediction because of both high sensitivity and specificity. In contrast, we found no significant differences for the cut-off points for HbA2 as a β-thalassemia predictor that was independent of gender and pregnancy. Therefore, we recommend a 3.24% cut-off points of HbA2 for β-thalassemia with high sensitivity and specificity. Our recommended cut-off points may lead to improvements in the accuracy of thalassemia screening in the reproductive age population in Chongqing. Nevertheless, an implementation of the pregnancy-specific cut-off points requires a long-term observation to verify its efficacy.

Three α gene deletion types (such as --SEA, -α4.2, and -α3.7), 3 α gene mutants (such as HbCS, HbWS, and HbQS), and 17 common β gene mutants account for 95% of thalassemia genotypes in the Chinese population.^23^ Among patients with α-thalassemia, there were mainly 3 α gene deletion types, while the most common mutation type in β-thalassemia carriers was *D17* (*HBB: c. 52A>T*), which was similar to previous reports.^24,25^

### Study limitations

This study was limited to the Chongqing area. It only established the reference range and diagnostic value, and it lacked a verification for the population. Future research will further verify the application value.

This study recommended the reference interval and critical value of HbA2 and HbF for the screening of thalassemia in Chongqing, China. The sample size was considerable, which truly reflected the regional characteristics of thalassemia. The established values for the screening were suitable for the population in this region, especially pregnant women.

In conclusion, this study provided valuable data to improve screening efficiency and further contributes to the prevention and reduction of thalassemia in pregnant women.
